# Rare Case of a Giant Left Atrium With Cerebrovascular Accident

**DOI:** 10.7759/cureus.14698

**Published:** 2021-04-26

**Authors:** Antoine El Khoury, Marc Achkar, Samer Nasr

**Affiliations:** 1 Cardiology, University of Balamand, Faculty of Medicine and Medical Sciences, Beirut, LBN; 2 Cardiology, Mount Lebanon Hospital, Beirut, LBN

**Keywords:** giant left atrium, atrial fibrillation, enlarged left atrium, mechanical valve, cerebrovascular accident, stroke

## Abstract

Left atrium enlargement is very common in patients with valvular heart disease and atrial fibrillation but an extremely dilated left atrium is a very rare condition and rarely reported in the literature. It is a risk factor for ischemic cerebrovascular accidents due to blood stasis as the cavity diameter increases. We are reporting a case of rarely seen severely dilated left atrium with a normal functioning prosthetic mechanical mitral valve with a cerebrovascular accident on anti-vitamin K and aspirin. The patient had a left atrium diameter of 12.7 cm, an area of 200 cm square, and a volume of 2000 cc. We elected to keep the international normalized ratio (INR) slightly above the therapeutic range in order to decrease the risk of ischemic events. It might be necessary to do the same for patients with a similar condition to decrease the stroke rates.

## Introduction

The left atrium is an important structure in the heart, as it receives oxygenated blood from the lungs by the pulmonary veins and participates in left ventricular filling. When opposed to high afterload and high pressure or when the heart electrical activity is irregular in case of atrial fibrillation, the left atrium will increase in size to compensate for the high afterload [1]. This occurs in cases of diastolic dysfunction, hypertension, and valvular heart disease due to an increase in left ventricular pressure and is a risk factor for heart failure and cerebrovascular accidents [2-4].

## Case presentation

**Figure 1:**
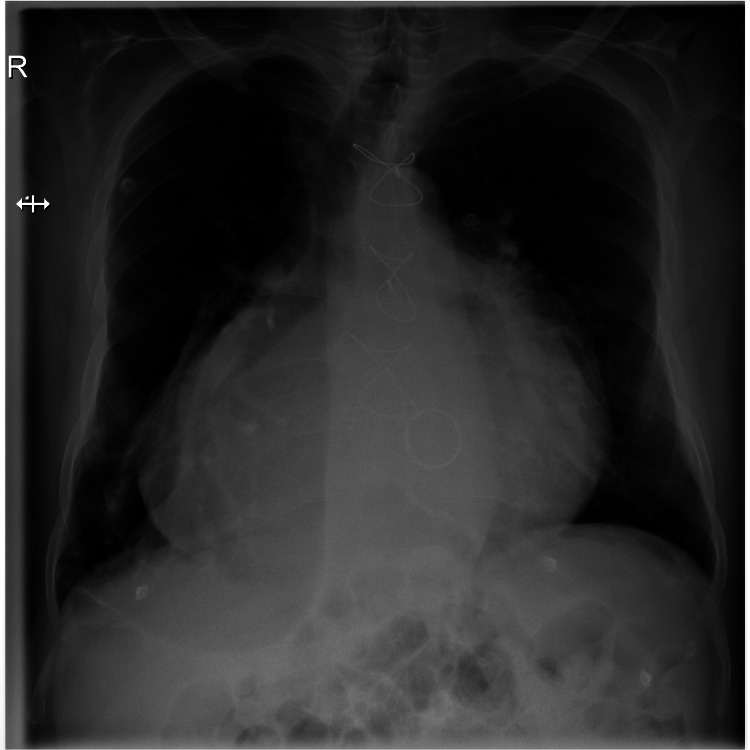
Chest X-ray showing massive cardiomegaly

Our patient was a 60-year-old male with a history of long-standing hypertension, dyslipidemia, and permanent atrial fibrillation. He underwent mechanical mitral valve replacement 10 years ago with no major complications. His medications were bisoprolol, aspirin, warfarin, spironolactone, and furosemide. He presented for acute onset of slurred speech and right upper limb weakness associated with hypertension (175/120 mmHg), with a heart rate of 75 beats per minute. ECG showed atrial fibrillation with normal ventricular rate and all laboratory tests were within normal range but he had a subtherapeutic international normalized ratio (INR) of 2.1. Chest X-ray showed massive cardiomegaly (Figure 1).

An urgent CT of the brain was done and showed no acute hemorrhage but showed bilateral hypodensities within the pons, which were slightly edematous, likely related to subacute/chronic ischemic events. A 5 mm hyperdensity was also seen in the distal aspect of the basilar artery and raised the possibility of a small thrombus.

**Figure 2:**
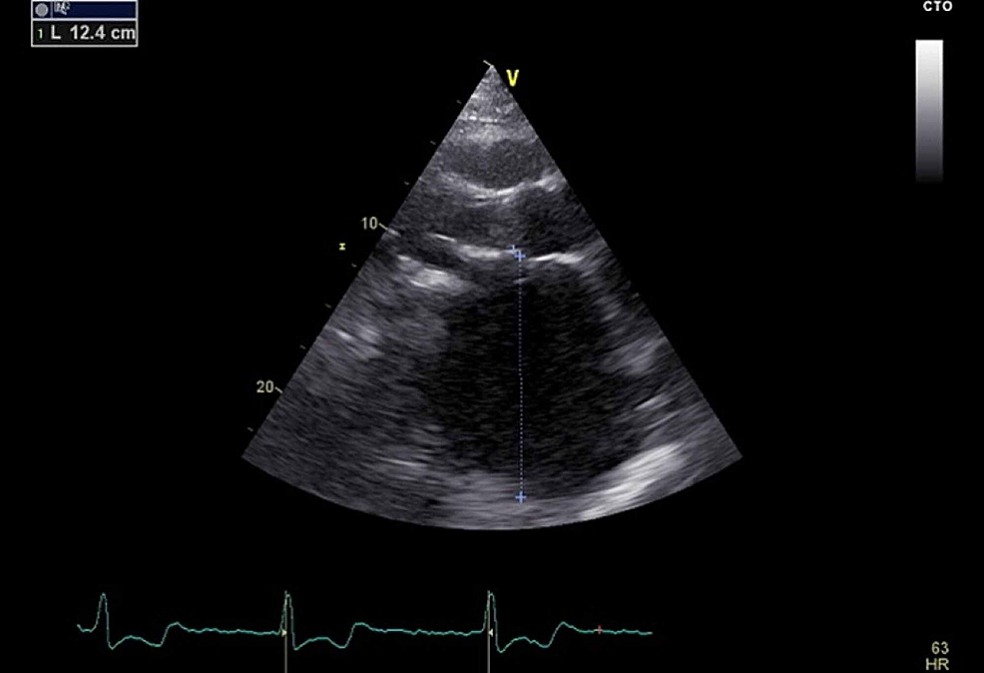
Echocardiography image of the left atrium (LA diameter) on the parasternal long-axis view

**Figure 3:**
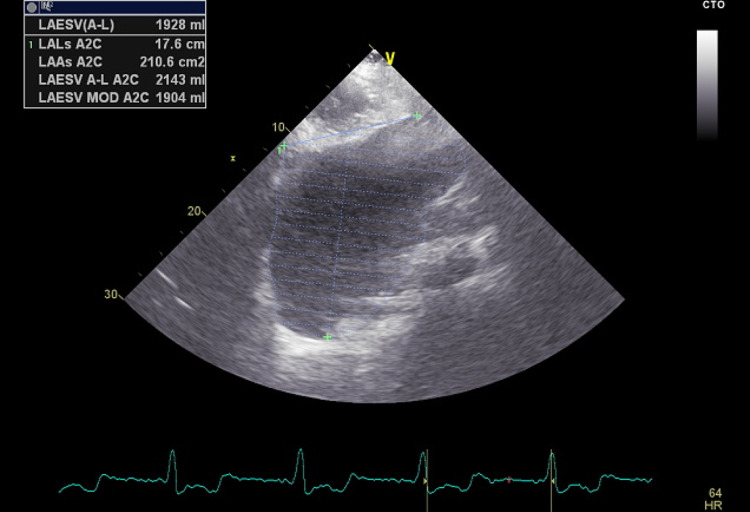
Echocardiography image of the left atrium (LA area)

**Figure 4:**
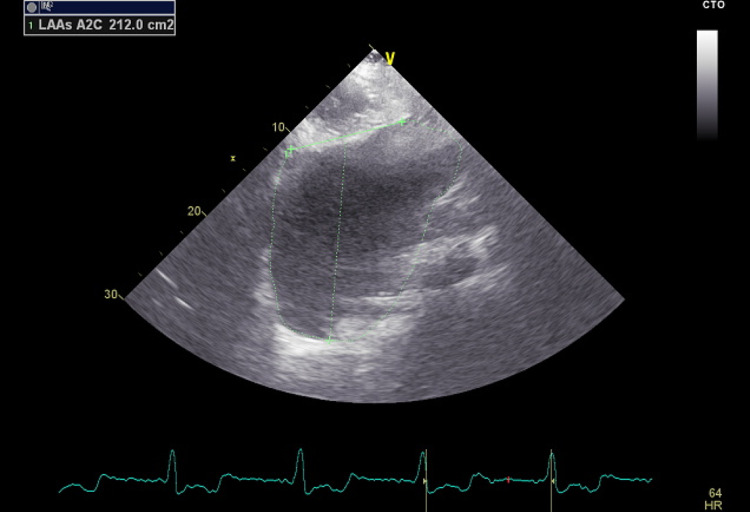
Echocardiography image of the left atrium (LA volume)

The patient was placed on enoxaparin and warfarin with a notable improvement of his symptoms in less than 24 hours and minimal residual weakness and slurred speech. He was scheduled for transthoracic echocardiography to assess his cardiomegaly and valve function and to look for a possible source of his brain embolic event. He had appropriate valve function but his left atrium was severely enlarged with a diameter of 12.4 cm and an area of 200 cm square and a volume of 2000 cc (Figures 2-4).

## Discussion

According to the American Society of Echocardiography, left atrium enlargement can be classified as mild, moderate, or severe. Diameters of 4.1-4.6 cm in men or 3.9-4.2 cm in women are considered as mild enlargement, whereas diameters of 4.7-5.1 cm in men and 4.3-4.6 cm in women are classified as moderate and diameters of ≥5.2 cm in men or ≥4.7 cm in women as large [5]. The terminology of huge left atrium is for diameters larger than 6 cm, and a left atrium with a diameter of more than 12 cm is very rarely reported in the literature. In addition to that, volumes above 2000 cc are very uncommon [6]. The causes of left atrium enlargement include mitral stenosis or regurgitation, rheumatic valve, left ventricular failure, hypertension, left ventricular diastolic dysfunction, aortic stenosis, and atrial fibrillation [7-8].

The determination of left atrial size by echocardiography is an acceptable way of determining prognosis and cardiovascular outcomes such as heart failure and strokes due to the increased risk of thrombus formation [9]. Left atrial enlargement is associated with an increased risk of atrial fibrillation, which in turn increases the risk of cerebrovascular events and recurrent strokes [10]. This relation was found to be significant in both men and women as previous studies have shown [5,11].

An enlarged left atrium is associated with a decreased ability of the heart to pump blood and is an independent risk factor for atrial fibrillation, and it was found that 19% of patients with an enlarged left atrial cavity had atrial fibrillation. Different studies showed that for every increase in LA diameter of more than 0.5 mm, we had a 39% increase risk of atrial fibrillation [12]. Atrial fibrillation is also a risk factor for left atrial enlargement due to impaired ventricular filling and impaired cardiac output and can result in heart failure if chronic and uncontrolled [13]. Valvular dysfunction, mainly mitral valve diseases or impaired prosthetic mechanical mitral valve, was associated with big LA sizes, but note that huge left atrial diameters were also observed in patients with prosthetic valves that had normal function [14]. Atria of this size can cause compression of surrounding structures, leading to dysphagia by compressing the esophagus, hoarse voice by compressing the recurrent laryngeal nerve, and respiratory depression due to lung compression [14].

The patient we presented developed an ischemic brain event despite being on warfarin and aspirin. The giant left atrium size increases the risk of ischemic brain events. In that case, we aimed to keep the INR level slightly above the therapeutic range in order to avoid similar future events with more frequent INR follow-ups. In addition, aspirin was replaced by clopidogrel because it was shown to be superior to aspirin in decreasing the risks for recurrent strokes [15]. Clopidogrel was also found to be superior to aspirin in reducing the risks for bleeding events [15]. In a different study, clopidogrel was found to be superior to aspirin in reducing gastrointestinal bleeding events only [16]. Clopidogrel and warfarin were continued together because it was shown that combining vitamin K antagonists (VKA) and antithrombotic therapy in patients with mechanical valves was superior to VKA alone [17-19]. There was a significant reduction in mortality rate and thromboembolism in the combination group with a moderate increase in bleeding events [17-18].

## Conclusions

Our case illustrates a rarely seen left atrial size on a mechanically well-functioning valve with a cerebrovascular accident. The differential diagnosis on presentation can be misleading (pericardial effusion, mediastinal mass, or mechanical valve dysfunction ). We elected to keep the INR above 3.0 and replaced aspirin with clopidogrel to decrease the risk of gastrointestinal bleeding and ischemic brain events. The combination of VKA and antithrombotics was also shown to decrease the risk of ischemic events, with a moderate increase in bleeding events. It might be warranted to do the same in the future for patients with extremely large left atrial size prior to the occurrence of a cerebrovascular ischemic accident.
